# Impact of anti-epileptic drug choice on discharge in acute traumatic brain injury patients

**DOI:** 10.1007/s00415-020-09769-5

**Published:** 2020-03-04

**Authors:** Lauren Harris, Sofie Hateley, K. T. Tsang, M. Wilson, B. M. Seemungal

**Affiliations:** 1 Neurolosurgical Department, Imperial College Healthcare NHS Trust, Charing Cross Hospital, London, UK; 2 Neurology Department, Imperial College Healthcare NHS Trust, London, UK; 3 Neuro-Otology Unit, Division of Brain Sciences, Imperial College London, London, UK

**Keywords:** All clinical neurology, All epilepsy/seizures, Anti-epileptic drugs, Prognosis

## Abstract

**Background:**

Anti-epileptic drug (AED) prophylaxis in the first-seven days post-traumatic brain injury (TBI) is known to reduce seizure frequency acutely. AED efficacy is equivalent; therefore, choice of AED may rest with their side-effects. We hypothesise that AEDs that impair balance will prolong recovery, shown by a longer hospital stay. We compared length of hospital stay (and reported dizziness) in TBI patients receiving the commonest AEDs used in our TBI patients, Phenytoin (which may cause imbalance), and Levetiracetam (which does not affect balance).

**Method:**

A retrospective observational study was performed on TBI patients admitted to a Major Trauma Unit between October 2013 and June 2018. 100 of 278 patients treated with phenytoin or levetiracetam monotherapy for seizure prophylaxis were included. The inclusion criteria of admission Glasgow Coma Score of 14 or more and length of stay less than 3 weeks minimised confounding variables such as non-ambulant patients. Length of hospital stay and incidence of dizziness were assessed.

**Results:**

The length of hospital stay was longer for patients on Phenytoin versus Levetiracetam, i.e., 10.74 vs. 7.58 days (*p* = 0.015; unpaired, two-sided *t* test). Dizziness reported by patients on phenytoin was 24% and levetiracetam was 8% (*p* = 0.018; Chi-squared test).

**Conclusion:**

In this cohort, using Phenytoin for acute TBI, seizure prophylaxis was associated with longer length of stay and more dizziness compared to Levetiracetam. Given their equivalent AED efficacy in acute TBI seizure prophylaxis, our data suggest that Levetiracetam is preferable to Phenytoin for early seizure prophylaxis in TBI. This requires evaluation in larger, prospective studies.

## Background

Worldwide, more than 50 million people have a traumatic brain injury (TBI) each year, and it is the leading cause of death and morbidity in young adults. [1] In the UK, 1.4 million people attend the emergency departments with a recent head injury and 200,000 are admitted annually.[2] Of these, 20% have features of skull fractures or brain injury and 28,000 die [2].

The use of AEDs for TBI is a point of contention. However, it is well recognised that early post-traumatic seizures, i.e., seizures occurring in the first-week post-TBI, affect 12% of severe TBI cases (Glasgow Coma Scale Score < 8), and is reduced to 3.6% with Phenytoin prophylaxis.[3, 4] Early seizure prevention reduces the risk of secondary brain injury, which can cause brain herniation and death.[5, 6] Additionally, early seizures are a risk factor for post-traumatic epilepsy, which may be refractory to treatment.[4, 7]

AEDs may be used to prevent early post-traumatic seizures in patients at high risk for seizures following head injury [4, 6]. Current guidelines for post-TBI seizure prophylaxis focus on seizure control efficacy, and Phenytoin and Levetiracetam are commonly used [5, 6, 8, 9]. The guidelines state that ‘Phenytoin is recommended to decrease the incidence of early post-traumatic seizures, when the overall benefit is felt to outweigh the complications associated with such treatment’ [6]. Historically, Phenytoin was the AED of choice; however, due to its complications, Levetiracetam is increasingly favoured [9]. Phenytoin and Levetiracetam possess similar efficacy; however, Phenytoin may cause vestibular dysfunction in the form of imbalance and dizziness among other side-effects [5, 10].

Since safe walking is an important component of recovery post-TBI, we hypothesised that Phenytoin may impede recovery and, therefore, increase time-to-discharge compared to Levetiracetam, in patients receiving post-TBI AED prophylaxis in a major UK trauma centre. We aim to assess the impact of AED on length of hospital stay and the incidence of dizziness in patients with TBI.

## Method

A retrospective observational study was performed at a Major Trauma Unit, located in a Trauma Centre in London covering a population of circa 4 million. All patients admitted to the Major Trauma Unit following a TBI who were treated with Levetiracetam or Phenytoin monotherapy for early seizure prophylaxis between October 2013 and June 2018 were assessed for the study. Patients were included if they had an admission Glasgow Coma Score (GCS) of 14 or more, and a length of stay less than 3 weeks. Exclusion criteria were patients on more than one anti-epileptic medication, or patients with known seizure disorders. The tight selection criteria were used in an attempt to control for the type of head injury and clinical picture of the patient population.

Patients were identified from daily lists kept by the trauma unit, and a retrospective medical record review was performed in accordance with the local institutional review board’s approvals. Data were initially analysed to identify the trend in prescribing patterns of anti-epileptic medications and to compare the length of hospital stay of all patients treated with either Phenytoin or Levetiracetam.

We recorded patient demographics, Glasgow Coma Score (GCS) on admission, length of treatment, dizziness, complication rates, surgical input, and length of ITU stay. Our two primary outcomes were: (1) time-to-discharge and (2) reported incidence of dizziness, in particular illusory self-motion. Every patient was originally assessed by the dedicated trauma therapy team. Patients with ‘dizziness’ had a lying–standing blood-pressure measurement and an electrocardiogram to rule out cardiovascular causes, and serum electrolytes and liver function tests including ammonia to rule out encephalopathy. When clinical suspicion warranted, patients received a transthoracic echocardiogram to investigate for cardiac-related causes. Patients were also reviewed by a senior consultant neurologist with an interest in vestibular dysfunction and trauma. He assessed the patient for ataxia, oculomotor, gait, and vestibular dysfunction to characterise the dizziness (e.g., for suspected cerebellar versus vestibular dysfunction, AED related versus benign paroxysmal positional vertigo following trauma). Electroencephalogram (EEG) was restricted to patients with a reduced GCS or clinical suspicion of seizure activity, and was not routinely used.

The data were analysed using simple descriptive statistics, unpaired two-tailed *t* test, and the Chi-squared test as appropriate. We Bonferroni corrected the significance level for two comparisons (*p* < 0.025). The inclusion criteria of admission GCS of 14–15 and a length of stay of less than 3 weeks were chosen as balance disturbance is only reliably assessed in ambulant patients, and additional confounds present with a more severe head injury (including surgery and complicated ITU admissions) can be reduced.

## Results

**Fig. 1:**
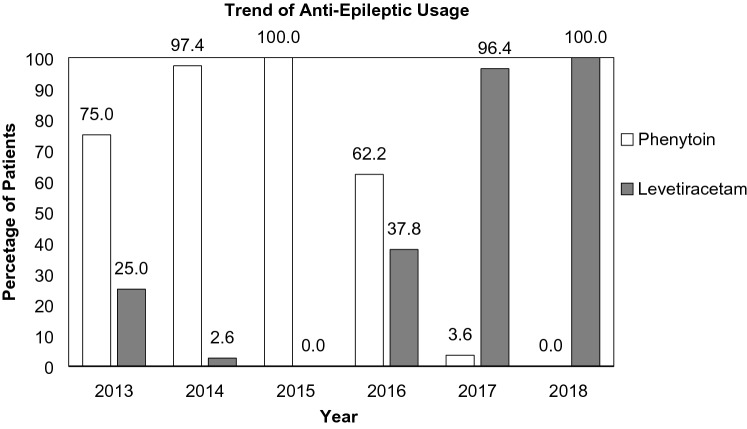
Trend in anti-epileptic usage over time: percentage of patients on Phenytoin or Levetiracetam from October 2013 to June 2018

During the period October 2013–June 2018, 278 patients were treated with either Phenytoin or Levetiracetam monotherapy. This was at standard dosing regimens as recommended by the British National Formulary and levels were monitored as appropriate. Of these, 100 patients met the additional inclusion criteria of an admission Glasgow Coma Score (GCS) of 14 or more and a length of stay less than 3 weeks. There has been a change anti-epileptic medication prescribed from Phenytoin to Levetiracetam (Fig. 1).

The 100 patients meeting our selection criteria, as expected, had fewer surgeries, ITU admissions, infections, and hospital complications, compared to the 178 patients not meeting the criteria. The baseline characteristics for Phenytoin versus Levetiracetam were comparable (all *p* > 0.05) for demographics, GCS on admission, ITU stay, duration of AED, surgery, or seizure (Table 1). The two groups were well matched for the incidence of skull fractures, subdural haematomas, extradural haematomas, and cerebral contusions. All patients were started on an AED due to clinical suspicion; whether that be due a seizure on admission or location of intracranial pathology on CT scan. They were all on routine doses (Phenytoin 300 mg once daily and Levetiracetam 500 mg twice a day unless difference due to weight or age consideration).

**Table 1:** Characteristics of patients treated with Phenytoin and Levetiracetam

	Phenytoin	Levetiracetam	*p* value
	*n* = 23	*n* = 77	
Demographics			
Age (years)	63.7 (22–89)	53.7 (15–95)	0.54
Male	18 (78.2)	57 (74.0)	0.68
GCS on admission			
GCS 14	11 (47.8)	32 (41.56)	0.60
GCS 15	12 (52.2)	45 (58.44)	
Complications			
ITU stay	1 (4.35)	1 (1.30)	0.36
Surgery	2 (8.70)	11 (14.29)	0.49
Seizure	1 (4.35)	4 (5.19)	0.87
Duration of AED	6 (1–43)	7 (1–89)	0.34

The demographical comparisons were not part of out a priori hypotheses (and were performed simply to show the equivalence between the two groups), and hence, they were not Bonferroni corrected (although they all were still non-significant). Data were given as mean and range, or frequency and percent as appropriate

Our main outcomes showed that Levetiracetam use was associated with a shorter length of hospital stay of 6 days (SD 5.23) versus 12 days (SD 5.87) for Phenytoin (*p* = 0.015; unpaired two-tailed *t* test). Dizziness was more common in Phenytoin-treated patients with 26% reporting dizziness versus 8% for Levetiracetam (*p* = 0.018; Chi-squared test). Phenytoin levels were routinely measured and dizziness did not appear to be concentration dependent. There was no significant difference in the incidence of post-traumatic benign paroxysmal positional vertigo (BPPV), which was found in one patient (4.3%) in the phenytoin cohort and two (2.6%) in the levetiracetam (*p* = 0.676; Chi-squared test). We Bonferroni corrected the significance level for two comparisons (*p* < 0.025).

Analysing patients with all admission GCS scores, where all appropriate data were documented (*n* = 177), there was no statically significant difference in the duration of AED or length of stay between Phenytoin and Levetiracetam. The median duration of AED was 7 for both groups. The median length of hospital stay was 12 days for Phenytoin (range 2–110) and 8.5 days for Levetiracetam (range 1–93). There was no correlation between admission GCS and duration of stay.

## Discussion

Our data suggest that Levetiracetam, used for early post TBI seizure prophylaxis, is associated with a shorter length of hospital stay and less dizziness, than Phenytoin. Dizziness, or the lack of it, may be one factor that contributes to recovery in patients following TBI.

A large randomised, double-blind, placebo-controlled trial identified a significant reduction in the incidence of early seizures in patients with severe TBI treated with Phenytoin, of 3.6% vs 14.2% [3]. A meta-analysis has shown that Phenytoin is beneficial in preventing early post-TBI provoked seizures [11]. Continuing Phenytoin results in an increase in late seizures [3]. It has been shown that Phenytoin and Levetiracetam are equally efficacious in preventing early seizures post TBI. This is supported by a body of literature, including well-conducted randomised-controlled trials [5, 12–15]. A meta-analysis of 1186 patients identified that the rate of early post-traumatic seizures was 5.4% with Levetiracetam and 3.4% with Phenytoin (no significant difference) [12]. Further meta-analyses in 2016 and 2018 identified no difference in efficacy between Levetiracetam and Phenytoin for post-TBI seizure prophylaxis, which is consistent with current guidelines [13, 16]. There is no evidence to support a reduction in the risk of late seizures or mortality using AEDs in TBI, and so a short course only is recommended [17].

Drug tolerability, including side effect profile and toxicity, is key in affecting compliance, patient rehabilitation, and length of stay. Phenytoin carries a high chance of side-effects, drug interactions, and harmful reactions including cardiotoxicity, anticonvulsant sensitivity syndrome, Stevens–Johnson syndrome, and tissue necrosis [14]. Phenytoin exhibits non-linear pharmacokinetics that necessitates therapeutic drug monitoring, whereas Levetiracetam is easy to dose, and does not require the serum level monitoring [18]. Levetiracetam has a lower rate of intolerable cognitive side-effects (10.4% versus 14.6% for Phenytoin) and gastrointestinal problems [14, 18]. Levetiracetam does have a propensity for neuropsychiatric complications with chronic use, although this is of lesser importance for short courses used in acute TBI [15, 18].

Studies, including our own, have shown a favourable safety profile for Levetiracetam over Phenytoin, with fewer complications during hospital admission [14–16]. In 2016, Xu et al. looked specifically at prophylactic AEDs post-TBI and reported a better safety profile, with a lower adverse drug reaction rate, for Levetiracetam versus Phenytoin [18]. Only eight observational studies and one randomised control study were included in the meta-analysis, with varying sample sizes [18].

Our study highlights the change in prescribing patterns from Phenytoin to Levetiracetam in our institution in recent years, consistent with the literature [9]. As expected, the rate of operations, ITU admissions, and other complications were higher with patients presenting with lower GCS. AEDs are recommended for severe TBI; however, in our unit, clinical practice varies considerably. Many patients with mild or moderate TBI receive AEDs, often due to a combination of clinical suspicion, seizures on admission, and the location of intracranial pathology on the CT scans, including frontal or temporal contusions. For this reason, sub-group analysis was performed focusing on patients presenting with a GCS of 14 and 15 to highlight any true differences pertinent to ambulant patients. This allowed us to assess ‘dizziness,’ including lying–standing blood-pressure and gait assessments. We speculate that this is why, the results looking at all admission GCS patients are not significant, but appear to show a trend towards favouring levetiracetam (median length of stay 8.5 versus 12 for phenytoin).

Dizziness, described as an illusory self-motion, is a well-known, common side effect of Phenytoin [10]. In addition, dizziness are a common sequelae in TBI with typically multiple causes of dizziness in a single patient [19]. Vestibular dysfunction in TBI is multifactorial [20]. Hence, TBI-related and drug-related dizziness can together retard rehabilitation and recovery. Our data suggest that Levetiracetam, which does not affect balance functioning, is better tolerated and associated with shorter admissions than Phenytoin in TBI patients with admission GCS of 14–15.

Our study has a number of limitations. Although our overall sample size is large, it is relatively small for sub-group analysis, particularly phenytoin. We expect, however, the strength of the significance to increase in a higher powered study. Due to the change in usage of AED with time, it is difficult to directly compare Phenytoin with Levetiracetam at equivalent time points. However, the department has not changed materially in the last 5 years, no new care protocols have been instated, and the medical and nursing team are largely consistent. Despite this, there is the possibility of two contradictory forms of bias. Once cardiovascular, cardiac, electrolyte, and encephalopathic causes of dizziness had been excluded, dizziness was evaluated subjectively and objective measurements of dizziness were not performed. If dizziness had been more present in the attending physician’s mind in the early years, this could lead to a recall bias and a change in AED could lead to less reporting with Levetiracetam treatment. Alternatively, if the physician developed more of an interest in vestibular dysfunction in TBI as time developed, this could lead to a confirmation bias and a subsequent increase in reporting. We suspect the latter is more likely, which would make the differences in dizziness greater when comparing AED.

This is a retrospective observational study; a randomised-controlled trial comparing Phenytoin versus Levetiracetam would provide higher quality data with more definitive findings. It is difficult to truly eliminate confounding variables, including co-morbidities, surgical requirement, social issues, and hospital acquired complications. We have attempted to do so by including only TBI cases with a length of stay less than 3 weeks. However, this means that we can only look at differences in length of stay when the overall duration was under 3 weeks. We recognise that our study was conducted on patients with a GCS 14–15, where dizziness could accurately be assessed, whereas the guidelines for AEDs in TBI are for those patients with severe TBI and GCS < 8. It is clear that AEDs are often prescribed for minor TBI based on clinician choice and it is this cohort that contributed [21]. Whether the AED was indicated at all in these patients was beyond the scope of this study. Specific examination findings, including neurological gait dysfunction and the rate of encephalopathy, were not included in this retrospective study. Despite this, we believe that our study represents an accurate comparison of Phenytoin and Levetiracetam and their impact upon dizziness and length of stay.

Recent studies have suggested that prophylactic Levetiracetam for patients with intracerebral haemorrhage was independently associated with lower cognitive function on follow-up [22]. We hope to assess the extent that prophylactic AEDs in TBI impacts long-term cognitive function in future work. This study provides preliminary data for larger studies looking at AEDs in post-TBI seizure prophylaxis and its impact upon duration of admission and the potential impact upon healthcare expenditure. Additionally, further study may help to assess the impact on post-discharge recovery. Dizziness post-TBI is an independent risk factor for delayed return-to-work in TBI [23]. Hence, AEDs that impact upon balance functioning may also affect return-to-work rates for the young patient group who are disproportionately affected by TBI.

## Conclusion

This study identified that TBI patients treated with Levetiracetam for post-TBI seizure prophylaxis have shorter inpatient admissions, with less balance disturbance, compared to patients on Phenytoin. Given that both AEDs have similar seizure prophylaxis efficacy, our data support the use of Levetiracetam over Phenytoin for acute and short-term, post-TBI seizure prophylaxis.

## Data Availability

Investigators have carefully documented data, methods, and materials used to conduct this research article. Any data not included in the article are available by request from investigators.
